# External RF-EMF alters cell number and ROS balance possibly via the regulation of NADPH metabolism and apoptosis

**DOI:** 10.3389/fpubh.2024.1425023

**Published:** 2024-08-09

**Authors:** Sheung-Ching Chow, Yang Zhang, Raymond W. M. Ng, Shu-Yuen Ron Hui, Ilia A. Solov’yov, Wing-Yee Lui

**Affiliations:** ^1^School of Biological Sciences, The University of Hong Kong, Pokfulam, Hong Kong SAR, China; ^2^Department of Electrical and Electronic Engineering, The University of Hong Kong, Pokfulam, Hong Kong SAR, China; ^3^Department of Electrical and Electronic Engineering, Imperial College London, London, United Kingdom; ^4^Institute of Physics, Carl von Ossietzky Universität Oldenburg, Oldenburg, Germany; ^5^Research Center for Neurosensory Science, Carl von Ossietzky Universität Oldenburg, Oldenburg, Germany; ^6^Center for Nanoscale Dynamics (CENAD), Carl von Ossietzky Universität Oldenburg, Oldenburg, Germany

**Keywords:** RF-EMF radiation, mid-range wireless power transfer, cellular responses, metabolism, reactive oxygen species

## Abstract

The influence of weak radio-frequency electromagnetic field (RF-EMF) on living organisms raises new concern because of the Industrial, Scientific, and Medical (ISM) frequency band at 6.78 MHz being promoted by the AirFuel Alliance for mid-range wireless power transfer (WPT) applications and product development. Human exposure to the RF-EMF radiation is unavoidable. In this study, we employed *in vitro* cell culture and molecular biology approach coupled with integrated transcriptomic and proteomic analyses to uncover the effects of RF-EMF on cells at molecular and cellular levels. Our study has demonstrated that weak RF-EMF is sufficient to exert non-thermal effects on human umbilical vein endothelial cells (HUVEC). Exposure of weak RF-EMF promotes cell proliferation, inhibits apoptosis and deregulates ROS balance. Alteration of several signaling pathways and key enzymes involved in NADPH metabolism, cell proliferation and ferroptosis were identified. Our current study provide solid evidence for the first time that the present safety standards that solely considered the thermal effect of RF-EMF on cell tissue are inadequate, prompt response and modification of existing Guidelines, Standards and Regulation are warranted.

## Introduction

Popularity of the Qi-certified wireless charging products in consumer electronics and manufacturing facilities has gained much attention. The AirFuel Alliance formed by a group of companies intends to expand the use of the wireless power transfer (WPT) technology for mid-range applications (1). The Qi standard has the safety features in place to minimize human exposure to radiofrequency electromagnetic field (RF-EMF), however, RF-EMF exposure seems unavoidable in mid-range wireless charging application. Given the fact that the present safety standards solely considered the thermal effect of RF-EMF on tissue, any potential non-thermal detrimental cellular effects remain largely unexplored (2). In the aspect of health concern, investigation of the effects of external RF-EMF on cells is warranted prior to the mid-range application of WPT technology.

The effects of RF-EMF influencing living organisms have been well documented. For instance, studies have been shown that a weak RF-EMF could affect the ability of migratory birds in perceiving the geomagnetic field, thus destroying the ability to navigate (3–5). It has been proposed that RF-EMF interacts with living matter through the radical pair mechanism. Transient radicals are abundant in cells which may interact with RF-EMF, resulting in alteration of the ROS balance (4–6). A computational approach has been previously proposed with detailed workflow to elucidate various molecular features of a radical pair system in response to RF-EMF (7). With the help of the computational approach, the potential effects of RF-EMF at certain frequencies on cells could be predicted accordingly.

So far, very limited experimental studies have been performed to explore the effects of external RF-EMF on cells, let alone their molecular regulatory mechanisms. Previous studies mainly focused on investigating RF-EMF of 900 MHz or higher and their respective effects on selected proteins and signaling molecules (8, 9). A recent study has shown that a weak external RF-EMF (10 μT at 7 MHz) could alter cell growth and the level of reactive oxygen species (ROS) (1), but the underlying molecular mechanisms remain elusive. It is well known that imbalance in the level of ROS in cells could alter various cellular functions and signaling pathways (10, 11). Therefore, it is of importance to explore the molecular mechanisms of how external weak RF-EMF, in particular for mid-range application, affects cellular processes.

To fill the research gap, human umbilical vein endothelial cells (HUVEC) were exposed to an external RF-EMF (10 μT at 6.78 MHz) for 72 h followed by RNA sequencing and proteomics analyses. RF-EMF of 6.78 MHz was specifically selected since it is one of the license-free Industrial, Scientific and Medical (ISM) frequencies originally decided for wireless communication and now it has been considered for WPT mid-range application. Through employing the integrated omics analyses and *in vitro* studies, we have depicted the altered downstream effects and the signaling pathways of the HUVEC cells. The results provide the direct study that demonstrates how external RF-EMF alters cell proliferation and ROS balance possibly via the activation of key pathways involved in NADPH metabolism and apoptosis.

## Materials and methods

### Cell culture

Human Umbilical Vein Endothelial Cells, HUVEC, were obtained from Lonza (Basel, Switzerland). Cells were cultured in Ham’s F-12 (Sigma-Aldrich, St. Louis, MO, United States) supplemented with 20 μg/mL endothelial cell growth (Sigma-Aldrich), 18 units/mL heparin (Sigma-Aldrich), 50 unit/ml penicillin–streptomycin (Hyclone, Boston, MA, United States) and 10% (v/v) fetal bovine serum (Hyclone) in a humidified incubator at 37°C supplied with 5% CO_2_. Cells were passaged with 0.05% trypsin–EDTA (Sigma-Aldrich) when the confluence reached 90%.

### Magnetic field chamber and cell culture

A magnetic field chamber with controllable amplitude and frequency was set up inside a humidified CO_2_ incubator. A pulse/ function generator (Hewlett Packard model number: 8116A) was used to produce a sinusoidal signal at 6.78 MHz and this signal was amplified by a power amplifier (Amplifier research, model number: 100A250A). When the signal passed through a coil made of litz wire, the excitation current was at 100 mA (measured by Agilent Technologies, model number: DSO-X 3024A), which is equivalent to a magnetic field of 10 μT.

For cell culture condition, HUVEC (2.5 × 10^4^ cells/well) were seeded on 24-well plates and cultured in humidified incubator for 24 h prior to RF-EMF exposure. RF-EMF-exposed HUVEC were incubated on the coil, which continuously generates the desired RF-EMF (10 μT at 6.78 MHz), for another 72 h. Control cells were cultured in the same setting but without the RF-EMF. To minimize the change of environmental parameters, the incubator remained tightly closed during the whole incubation period.

### MTT assay

An equal number of HUVEC was placed in a 96-well plate and cultured as described in above section. After 72 h exposure to RF-EMF, MTT solution (250 μg/mL) were added into each well and cells were incubated for 2 h. The resulting formazan product was dissolved in DMSO (Sigma-Aldrich). The absorbance was measured at 570 nm using VICTOR multilabel plate reader (PerkinElmer, Waltham, MA, United States).

### TUNEL assay

HUVEC cells were seeded on the glass coverslip (triplicate per treatment) in a 24-well-plate and cultured in the incubator with/without magnetic field chamber for 72 h. Cells were stained using an *in situ* apoptosis detection kit (Roche, Basel, Switzerland) and then mounted with DAPI (Vector Laboratories, Inc., Burlingame, CA). 10 views/coverslip were randomly selected and examined.

### Cellular ROS detection assays

Cellular superoxide (O_2_^•-^) and hydrogen peroxide (H_2_O_2_) in HUVEC were detected using cellular ROS detection assay kit (Abcam, Cambridge, United Kingdom) and ROS-Glo™ H_2_O_2_ assay kit (Promega, Madison, WI, United States) respectively. HUVEC were cultured as described in cell culture section. HUVEC were cultured in humidified incubator overnight followed by exposure of RF-EMF (10 μT at 6.78 MHz) for 24 h. For O_2_^•-^ assay, cells were stained with ROS red working solution for 1 h. Fluorescence intensity at 605 nm was measured using VICTOR multilabel plate reader. For H_2_O_2_ assay, H_2_O_2_ substrate was added to the culture medium after 18 h RF-EMF exposure and cells were further incubated for 6 h with RF-EMF exposure. ROS-Glo™ detection solution was then added before detection. Luminescence was recorded with VICTOR multilabel plate reader.

### Quantitative PCR

Total RNA was isolated from HUVEC with or without 72 h exposure of RF-EMF using TRIZOL reagent (Thermo Fisher Scientific, Waltham, MA, United States). cDNA was synthesized with high-capacity cDNA reverse transcriptase kit (Thermo Fisher Scientific). Reverse-transcribed products were analyzed with power-up SYBR green master mix (Thermo Fisher Scientific) for quantitative PCR by StepOnePlus™ Real-Time PCR system (Thermo Fisher Scientific) as previously described (12, 13). Primer pairs used in this study are listed in Table 1. Glyceraldehyde-3-phosphate dehydrogenase (GAPDH) was used for normalization. The specificity of the fluorescence signal was confirmed by melting-curve analysis. The expression level of the target genes was determined by the 2^−∆∆Ct^ method.

**Table 1:** Primer pairs used for real-time quantitative PCR.

	Primer sequence (5′–3′)			Product size (bp)
Gene	Forward	Reverse	Position	
ACOX1	GGCGCATACATGAAGGAGACCT	AGGTGAAAGCCTTCAGTCCAGC	1,032–1,144	112
AKR1C1	GCAAGTCAAAAGACATTGTTCTGG	TTGCCAAGGCACAAAGGACTGG	616–736	120
AKR1C3	CCGAAGCAAGATTGCAGATGGC	GTGAGTTTTCCAAGGCTGGTCG	194–307	113
CA8	CTCCACTCTGTTTGGCAGCATTG	GGAGGATTTCAGTCACAGCCTTC	437–559	122
ETFA	TGGCGGTAGTGCCAGTTCAGAA	CTCTCCACTCTTCAAGCCTCGA	536–687	151
GJA5	TAGGCAAGGTCTGGCTCACTGT	GAAAGCCTGGTCGTAGCAGACA	61–210	149
ME1	GGATAAAGCCGACCCTCTTCCA	TGGAAGAGGGTCGGCTTTATCC	1,377–1,502	125
MYLK2	GCTGTATGCAGCCATCGAGACT	ATGGTGTCCACCTCGGTCAGAT	1,031–1,151	120
NOS3	GAAGGCGACAATCCTGTATGGC	TGTTCGAGGGACACCACGTCAT	1,553–1,688	135
NOSTRIN	AGACCTCAACCCAGCCATCCTT	GAAGAACCTGGATTGCTCTGCC	891–1,052	161
TGFBI	GGACATGCTCACTATCAACGGG	CTGTGGACACATCAGACTCTGC	1,022–1,171	149
TXNRD1	GTTACTTGGGCATCCCTGGTGA	CGCACTCCAAAGCGACATAGGA	496–616	120
UQCRFS1	CCTGTGTTGGACCTGAAGCGG	CAGAGAAGTCAGGCACCTTGATG	120–268	148

### RNA sequencing

Total RNA was isolated from HUVEC with or without 72 h exposure of RF-EMF using TRIZOL reagent and purified with a RNeasy Mini kit (Qiagen, Germantown, MD, United States). Samples with RNA integrity numbers >8 were subjected to RNA sequencing performed by the Center for PanorOmic Sciences, CPOS (Li Ka Shing Faculty of Medicine, The University of Hong Kong). Four biological replicates were included for each condition. Sequencing reads were filtered for adapter sequence and low-quality sequence followed by retaining only reads with read length ≥ 40 bp. Sequencing reads were filtered for rRNA sequence and remaining reads were used for downstream analysis. Reads were mapped to the human genome GRCh38 (from GENCODE) using STAR (Ver. 2.5.2, Alex Dobin), while differentially expression analysis was done using EBSeq (Ver. 1.18.0, Ning Leng and Christina Kendziorski). The gene list ranked by posterior fold change and false discovery rate was subjected to Partek to identify biological processes and molecular functions (as defined by the Gene Ontology Consortium) showing a significant gene set enrichment.

### LC–MS/MS proteomics

HUVEC (1 × 10^6^ cells) with and without 72 h exposure of RF-EMF were pelleted. Proteins were extracted, processed and digested with EasyPep™ Mini MS Sample Prep Kit (Thermo Scientific), and were finally dissolved in 0.1% formic acid for LC–MS/MS analysis. Quantitation of peptides and LC–MS/MS analysis were performed by CPOS.

Eluted peptides were analyzed with nanoelute UHPLC, which was coupled with Bruker timsTOF pro mass spectrometer. Peptide mixture was loaded onto an Aurora C18 UHPLC column (IonOpticks, Australia). Chromatographic separation was carried and MS data was collected. The raw data were searched using the Andromeda algorithm against Mouse UniProt FASTA database (May 2020) containing 55,398 entries & Human UniProt FASTA database (Apr 2020) containing 74,824 entries. Confident proteins were identified using a target-decoy approach with a reversed database, strict false-discovery rate 1% at peptide and PSM level. Proteins identified from both conditions were quantified using the peptide LFQ intensities and their ratio obtained were used for label free quantitation to calculate the fold change. Data visualization and statistical data analysis were performed by Perseus software version 1.6.13.0.

### Statistical analysis

For all assays, data were shown as the mean ± SD of ≥3 independent experiments. For all studies, student’s *t*-tests were performed by GraphPad Prism 10 (GraphPad Software, La Jolla, CA, United States).

## Results

### Weak RF-EMF (10 μT at 6.78 MHz) prevents cells from apoptosis and causes an increase in cell number

**Figure 1:**
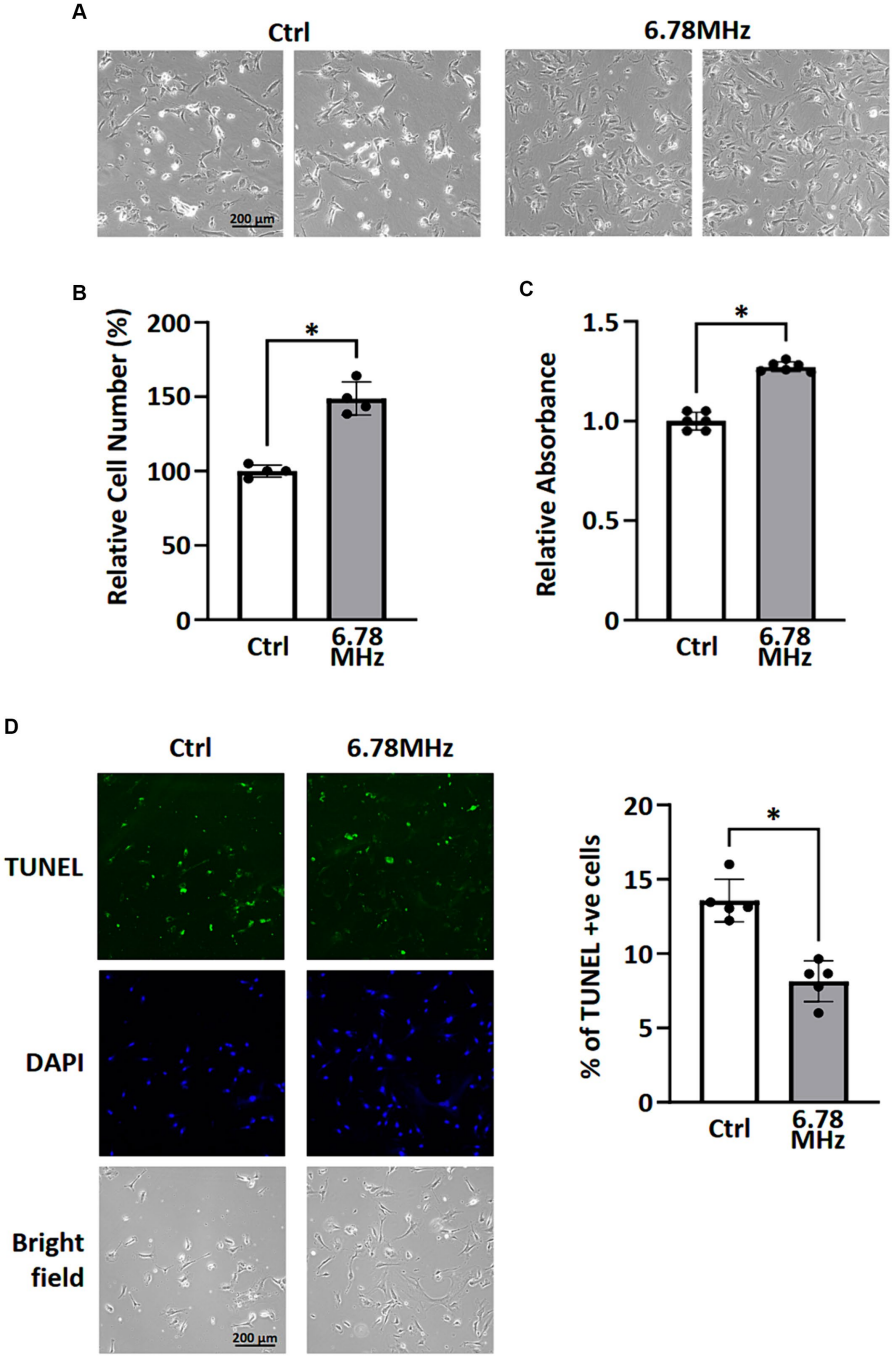
HUVEC cells were incubated in a culture chamber with or without the exposure of RF-EMF (10 μT at 6.78 MHz) for 72 h. After incubation, cells were harvested for morphological analysis **(A)**, cell counting **(B)**, MTT assay **(C)**, and TUNEL assay **(D)**. Data are presented as mean ± SD of three biological replicates. * indicates *p* < 0.05.

After 72 h exposure of the HUVEC to weak RF-EMF (10 μT at 6.78 MHz), an significant increase in viable cell number was observed (Figures 1A,B) compared to the control group, which was also in line with the result of MTT assay (Figure 1C). Figure 1A reveals that the number of dead cells in RF-EMF-exposed group was significantly fewer than that in the control one (Figure 1B). The TUNEL assay was therefore performed to validate cell apoptotic status. We found that the percentage of TUNEL-positive cells per total number of cells in culture was significantly reduced in RF-EMF-exposed cells, suggesting that RF-EMF prevents cells from apoptosis. Taken all results together, we confirmed that the exposure of the HUVEC to the weak RF-EMF could cause an increase in cell number which aligns with the previous report from other research group (1).

### RNA sequencing and LC–MS/MS-based identification of genes and proteins reflecting RF-RM field exposure

**Figure 2:**
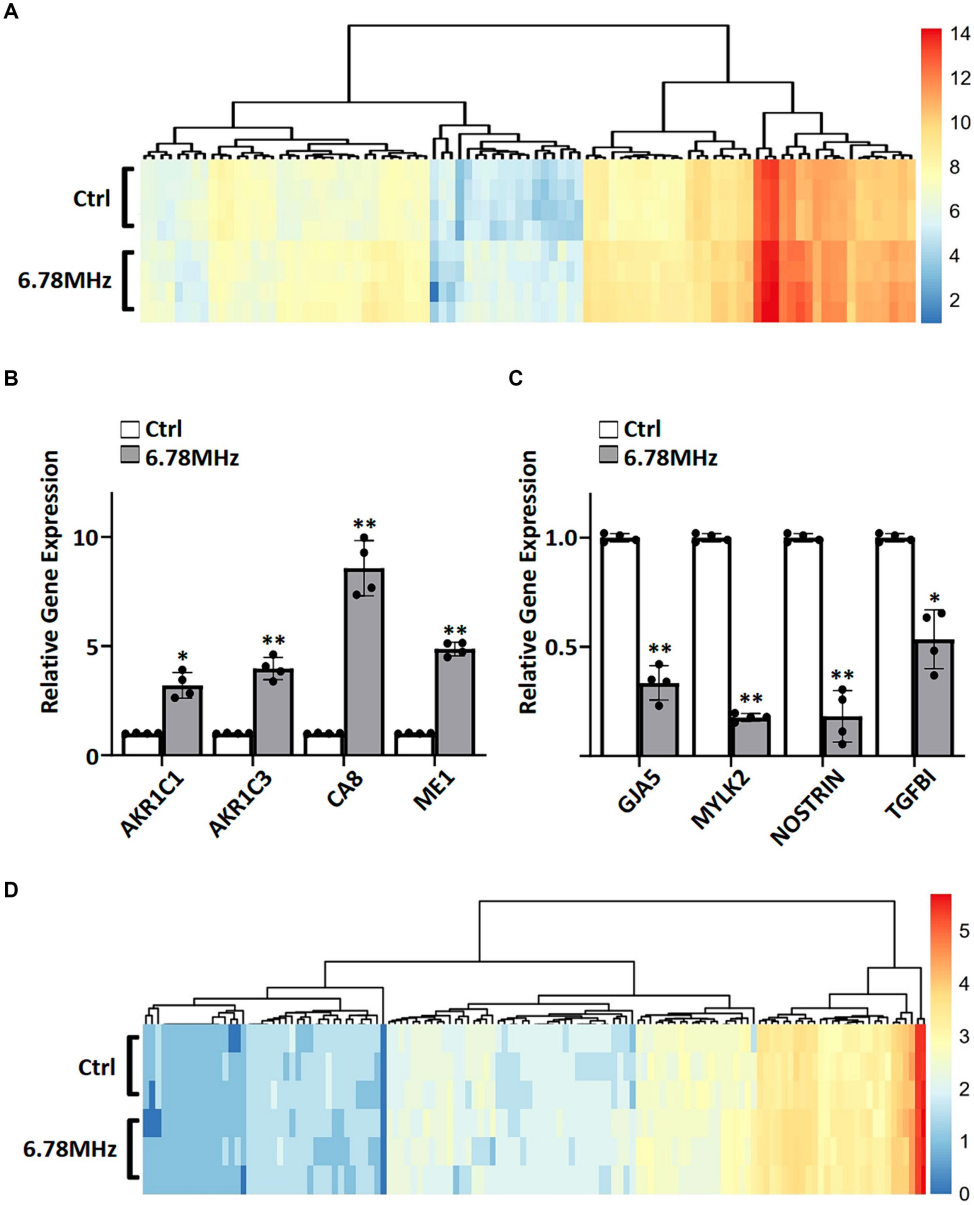
A weak RF-EMF alters transcriptomic and proteomic profiles in HUVEC cells. Heatmaps of fold change (log_2_) in expression for DEGs **(A)** and DEPs **(D)**. DEGs and DEPs were determined by comparing gene profiles of HUVEC exposed to 6.78 MHz EMF for 72 h vs. control. Quantitative PCR was performed to verify transcriptomics data. Relative expression of up-regulated **(B)** and down-regulated **(C)** genes are shown. GAPDH was included as an internal control. Relative expression was achieved by normalization to the amount of GAPDH using 2^−ΔΔCT^ method. Data is presented as mean ± SD of three biological replicates. * indicates *p* < 0.01 and ** indicates *p* < 0.001.

To gain insight into the genes and proteins that are affected or altered upon the exposure of the HUVEC to RF-EMFs, we carried out RNA sequencing on HUVEC cells with and without the exposure of weak RF-EMFs. Analyses of the 60,726 coding genes mapped in RF-EMF exposed cells and/or unexposed one (control) revealed that the 101 differentially expressed genes (DEGs) were identified using the criteria of *p* value <0.05 and ≥ 1.5-fold change (Figure 2A; Supplementary material S1). Altered genes with similar fold-change patterns were consistently observed in four separate biological samples from the control and RF-EMF-exposed group as shown in the heatmap (Figure 2A). Among the altered genes, we have selected some positive-regulated genes that are known to promote cell proliferation, reduce the oxidative stress and alter the NAD(P)/NAD(P)H homeostasis for qPCR validation (14, 15). For example, qPCR confirmed that the mRNA levels of carbonic anhydrase 8 (CA8), aldo-keto reductase family members including AKR1C1 and AKR1C3 and malic enzyme (ME1) were significantly increased (Figure 2B), which perfectly matches with the transcriptomic data (Figure 2A; Supplementary material S1). Similar to the positive-regulated genes, our qPCR validation on negative-regulated genes (Figure 2C) were also matched well with the transcriptomic data (Figure 2A; Supplementary material S1).

The liquid chromatography mass spectrometry (LC–MS/MS) tandem approach has identified 2,618 proteins in RF-EMF exposed cells and/or unexposed one. Among them, 136 differentially expressed proteins (DEPs) were identified using the criteria of *p* value <0.05 and ≥ 1.5-fold change (Figure 2D; Supplementary material S2). As shown in the heatmap, similar fold-change patterns of DEPs were consistently observed in three separate biological samples from the RF-EMF-exposed and control groups (Figure 2D).

### Transcriptomic and proteomic profiling indicates the effects of RF-EMFs on cellular processes and molecular functions

**Figure 3:**
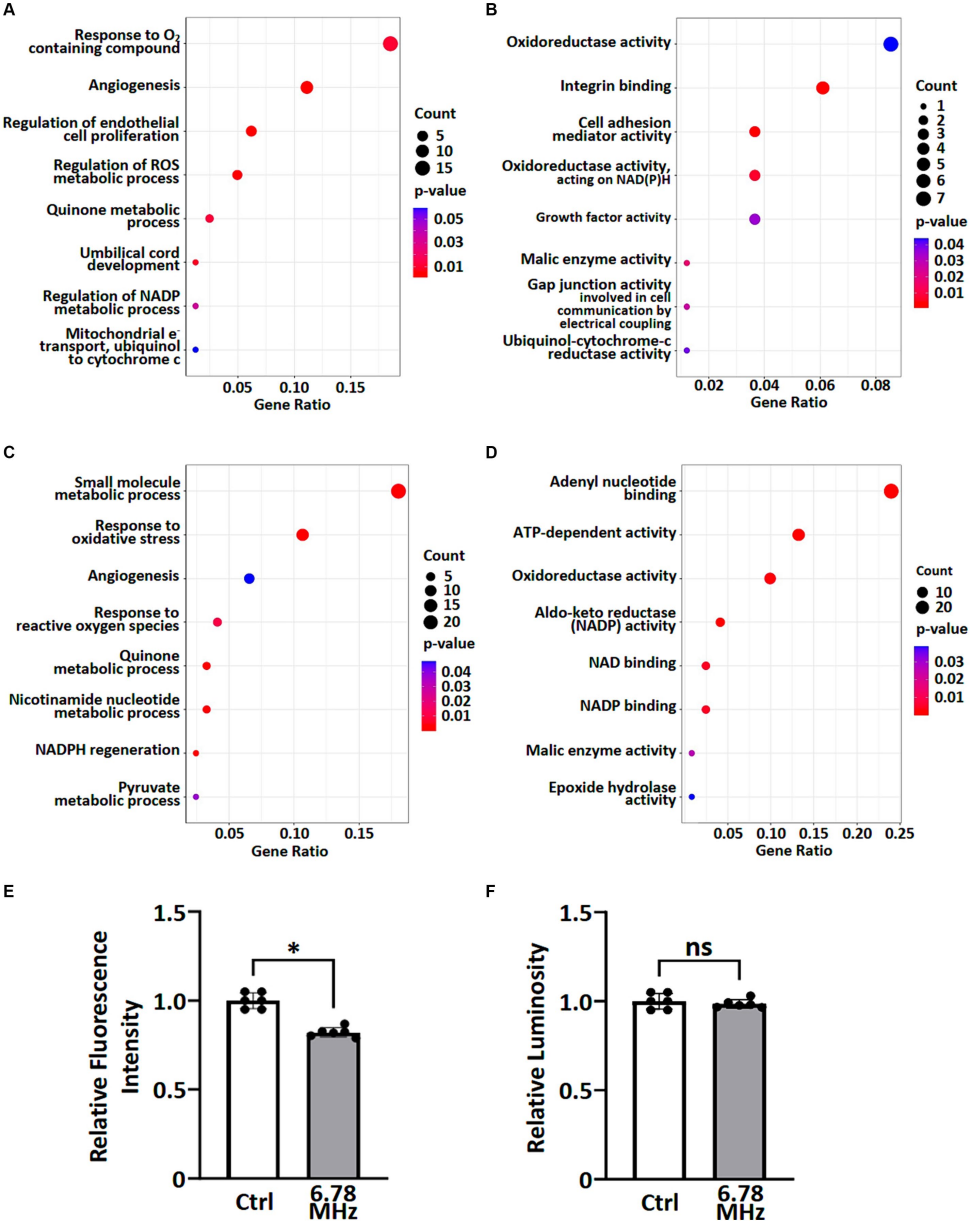
GO enrichment analysis of transcriptomic and proteomic data were performed and ROS metabolic processes were examined. A group of altered biological processes **(A,C)** and molecular functions **(B,D)** were selected and shown based on GO enrichment analysis of transcriptomics **(A,B)** and proteomics **(C,D)**. See Supplementary material S1, S2 for complete lists. Levels of superoxide (O_2_^.−^) **(E)** and H_2_O_2_ **(F)** in pertinent to ROS metabolic processes were examined using cellular ROS detection assays. Data is presented as mean ± SD of three biological replicates. * indicates *p* < 0.01; ns, not significant. Count indicates the number of altered genes/proteins identified in the specified process or function.

To understand the potential biological functions of altered genes and proteins, we classified the DEGs and DEPs according to gene ontology (GO) annotations of biological processes and molecular functions. Based on GO enrichment analysis of all DEGs and DEPs, the major altered GO terms linked to biological processes were response to ROS, NADP metabolic processes, quinone metabolic process and angiogenesis (Figures 3A,C). In term of molecular functions, the DEGs and DEPs were primarily enriched in oxidoreductase activity, aldo-keto reductase (NADP) activity and malic enzyme activity (Figures 3B,D).

To explore the effects of weak RF-EMF exposure on ROS metabolic processes as reported in Figures 3A–D, we examined the levels of superoxide (O_2_^•-^) and H_2_O_2_ using cellular ROS detection assays. It was found that the level of superoxide (O_2_^•-^) reduced significantly while the level of H_2_O_2_ remained steady after 24 h exposure of weak RF-EMF when compared to the control group (Figures 3E,F). Taken together, data obtained from the integrated omics analyses as well as our experimental studies on cell proliferation and ROS levels clearly suggest that weak RF-EMF exposure could affect cellular processes.

### Weak RF-EMF exposure alters numerous NADPH production pathways that are crucial for redox homeostasis and biosynthetic reactions in pertinent to rapid cell growth

**Figure 4:**
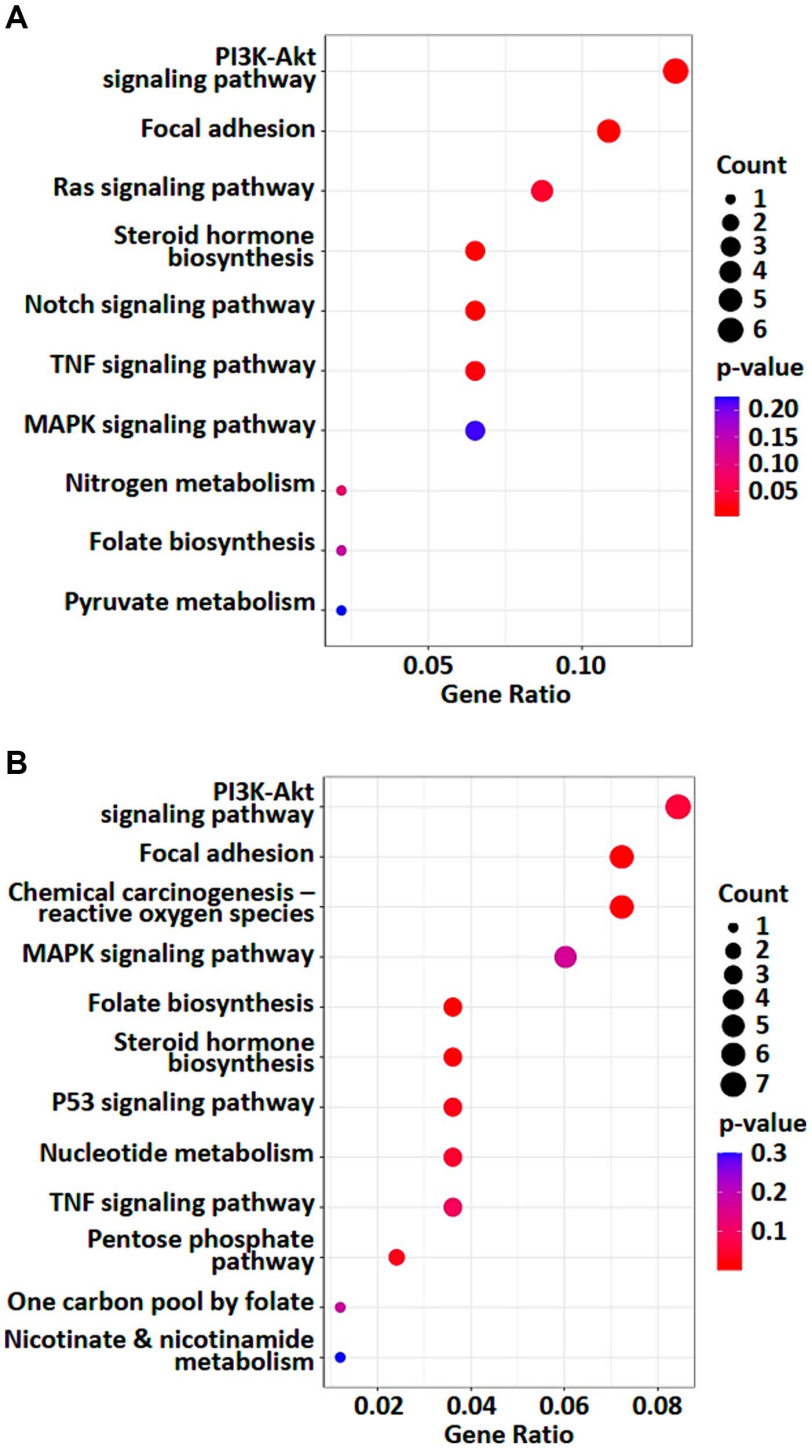
Enriched KEGG pathway analyses were performed. A list of selected enriched KEGG pathways based on analysis of transcriptomics **(A)** and proteomics **(B)**. Count indicates the number of altered genes/proteins identified in the specified KEGG pathway.

**Figure 5:**
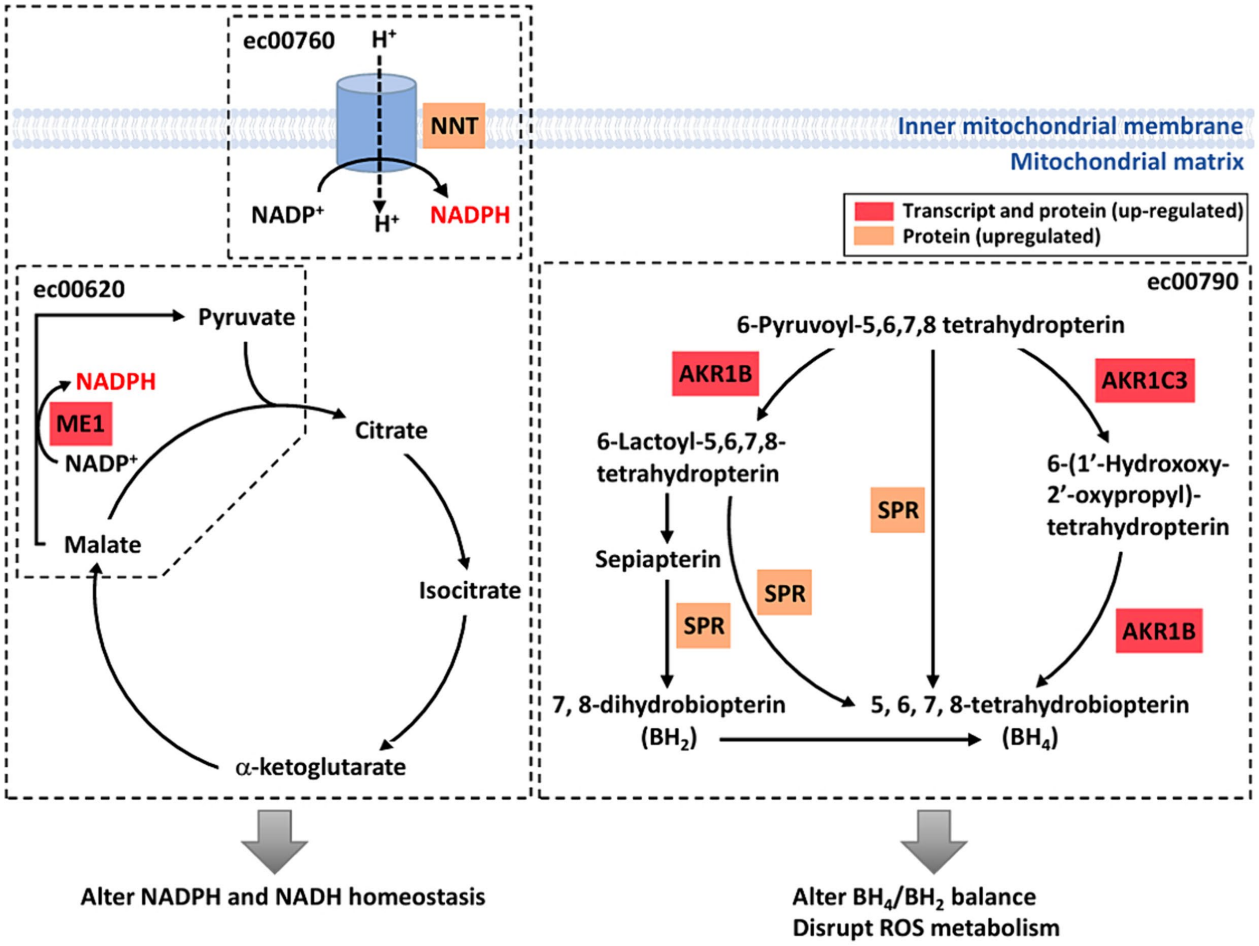
A diagrammatic drawing illustrating a regulatory mechanism of RF-RMF-triggered cell responses. DEGs, DEPs and altered signaling pathways were indicated in response to RF-EMF exposure. KEGG Pathways: pyruvate metabolism (ec00620), nicotinate and nicotinamide metabolism (ec00760), folate biosynthesis pathway (ec00790).

To investigate the effects of weak RF-EMF exposure, Kyoto Encyclopedia of Genes and Genomes (KEGG; http://www.genome.jp/kegg/) pathway enrichment analysis was performed using the deregulated genes and proteins identified in control and weak RF-EMF-exposed groups (16). Numerous canonical signaling pathways and genes including energy metabolism (including pyruvate metabolism and pentose phosphate pathway), focal adhesion signaling, folate biosynthesis and PI3-Akt pathway involved in the regulation of cell proliferation and cancer development are significantly enriched in both transcriptional (Figure 4A; Supplementary material S3) and translational (Figure 4B; Supplementary material S4) levels (Figure 5) (17, 18). Pyruvate metabolism (KEGG ec00620) is a canonical pathway that is directly related to NADPH/NADH production (Figures 4A,B). Apart from pyruvate metabolism (KEGG ec00620), NADPH homeostasis, in particular, nicotinate and nicotinamide metabolism (KEGG ec00760) via NNT was also altered at the translational level (Figure 4B). Apparently, genes and proteins involved in NADPH/NADH homeostasis has been perturbed in HUVEC cells via various molecular regulatory mechanisms upon weak RF-EMF exposure.

Apart from NADPH metabolism, pathway enrichment analysis also showed that folate biosynthesis pathway (KEGG ec00790) was altered at both transcriptional and translational levels (Figures 4A,B). In fact, folate biosynthesis pathway (KEGG ec00790) is not only a putative NADPH production pathway, but also a key pathway that regulates the tetrahydrobiopterin/dihydrobiopterin (BH4/BH2) balance. AKR1B, AKR1C and SPR proteins in folate biosynthesis pathway were found to be significantly upregulated in the weak RF-EMF exposed group and they were responsible for the production of BH4. A higher BH4/BH2 ratio is known to facilitate detoxification of ROS, thus reducing oxidative stress and promoting cell proliferation.

## Discussion

Unlike the short-range Qi-certified wireless charging products, human exposure to the AC electromagnetic field in mid-range WPT applications is unavoidable. Given that the current safety standards solely focus on RF-EMF radiation-induced thermal stress on tissue without taking any possible non-thermal detrimental cellular effects into consideration, an urgent and comprehensive investigation on its effects at cellular level is warranted to address any potential health concerns prior to the development of mid-range WPT application. In our current study, we unravel the effects of RF-EMF on human cells. The omics analyses and *in vitro* studies have demonstrated for the first time that 72 h of RF-EMF exposure (10 μT at 6.78 MHz) not only affects apoptosis and ROS balance, but also perturbs various key signaling pathways such as NADPH metabolism and PI3K signaling that are crucial in tumorigenesis.

Our study has shown that RF-EMF exposure could inhibit apoptosis and alter the ROS balance with a significant decrease in superoxide (O_2_^•-^) level in HUVEC. It is well-known that uncontrolled growth and apoptosis evasion are the hallmarks of all cancer cells regardless of the cause or type (19, 20). Also, oxidative stress contributed by ROS imbalance plays an essential role in the pathogenesis and progression of many diseases including cancer (21). The effects of RF-EMF on HUVEC indeed provides us some insights on the potential health concerns of the expansion of the mid-range wireless charging application where human exposure of RF-EMF is unavoidable.

Numerous evidence has shown that regeneration and maintenance of high level of cellular NADPH showed a strong implication in tumorigenesis. Altered NADPH homeostasis affects antioxidant system, apoptosis and ferroptosis in various pathological conditions such as cancer development and progression (22, 23). In general, tumour cells acquire high level of NADPH compared to normal cells (24). High level of NADPH not only helps supporting the biosynthesis of essential materials for rapid cell growth, but also promotes the generation of reduced forms of antioxidant molecules that power up the redox defense (25, 26). Our studies have confirmed that RF-EMF exposure modulates several pathways and enzymes that regulate NADPH level, ROS balance and inhibit apoptosis directly and indirectly. We therefore believe the weak RF-EMF exposure of mid-range WPT application do pose a potential health hazard issue that deserves attention.

Based on our studies, a regulatory model has been proposed to illustrate the molecular mechanisms and their interactions on how weak RF-EMF exposure affects cellular processes that could result in cancer development (Figure 5). Two important enzymes including malic enzyme 1 (ME1) and nicotinamide nucleotide transhydrogenase (NNT) that are involved in Krebs cycle and electron transport chain in pertinent to NADPH homeostasis were significantly increased upon weak RF-EMF exposure (Figure 5). In several cancer types, these two enzymes have found to be dysfunctional and are highly associated with cancer development and progression as well as prognosis. For instance, overexpression studies have found that ME1 promotes cancer growth and metastasis via NADPH homeostasis in various cancers including gastric cancer, basal-like breast cancer and lung cancer, etc. (27, 28). In adrenocortical cancer cells, it is found that NNT is upregulated and a high level of NNT thus activates anti-apoptosis pathways (29). NNT also regulates redox balance, resulting in promoting gastric cancer growth and metastasis (30). NNT can also prevent ROS-induced cytotoxicity in immune cells and adrenal tissues via its effect for detoxification of ROS by glutathione and thioredoxin pathways (31). The cellular responses such as enhancing cell proliferation and inhibiting apoptosis observed upon the exposure of weak RF-EMF in fact highly resemble the effects of upregulated ME1 and NNT on cancer cells, suggesting that ME1 and NNT are crucial regulators that mediate the biological effects of weak RF-EMF on cells. Since ROS and NADPH homeostasis are tightly associated with each other and in pertinent to cancer development. In-depth studies are warranted to identify the identification of key enzymes and regulators involved and to unravel the mechanisms of how RF-EMF alters ROS and NADPH homeostasis and their interplays result in apoptosis evasion.

AKR1B and AKR1C3, members of the aldo-keto reductase (AKR) enzyme family, were upregulated after the exposure of weak RF-EMF. Their primary role is to catalyse the NADPH-dependent conversion of aldehydes and ketones to primary and secondary alcohols. However, recent studies demonstrated that AKR1B and AKR1C3 are highly expressed in many human cancers including prostate, neuroblastoma and lung and they are crucial to regulate cell differentiation and cancer cell proliferation as well as to mediate drug resistance (32–37). More importantly, AKR1B, AKR1C3 and sepiapterin reductase (SPR) are crucial enzymes in the *de novo* and the salvage pathways of tetrahydrobiopterin (BH4) biosynthesis (38). Upregulation of these three enzymes after weak RF-EMF exposure could promote BH4 biosynthesis and cause a shift of BH4:BH2 ratio. Numerous studies have clearly demonstrated that AKR1Cs and BH4 synthesis are sufficient to exhibit antioxidant properties and to protect cells from ferroptosis (39, 40).

From integrated omics analysis, PI3K-Akt signaling and angiogenesis were found to be enriched significantly upon the exposure of weak RF-EMF. Key downstream effectors of PI3K-Akt pathway including PKN and CCND1 as well as vascular pro-angiogenic factor, vascular endothelial growth factor (VEGF), were significantly upregulated. PKN was found to contribute to the motility behaviour of human prostate cancer cells via the reorganization of actin filament (41) and CCND1 has been implicated in cancer progression in the aspects of cell proliferation, invasiveness and migratory behaviour in various cancers such as lung and breast cancer (42, 43). VEGF acts as mitogen in endothelial cells and promotes angiogenesis and cancer progression (44). Upregulation of pro-tumorigenic factors upon RF-EMF exposure indeed suggest RF-EMF for mid-range application pose a health risk. Further extensive studies are required to unravel the underlying mechanism.

Our current studies clearly demonstrated that cells are sensitive to weak RF-EMF. Various key regulators involved in NADPH metabolism, cellular metabolic activity and apoptosis were altered upon the exposure of RF-EMF. Our studies therefore provide solid evidence that the present safety standards solely considered the thermal effect of RF-EMF on cell tissue are inadequate, prompt response and modification of existing Guidelines, Standards and Regulation are warranted.

## Data availability statement

The original contributions presented in the study are included in the article/Supplementary material, further inquiries can be directed to the corresponding author.

## Ethics statement

Ethical approval was not required for the studies on humans in accordance with the local legislation and institutional requirements because only commercially available established cell lines were used.

## Author contributions

S-CC: Data curation, Formal analysis, Investigation, Writing – original draft, Writing – review & editing. YZ: Data curation, Formal analysis, Investigation, Software, Writing – original draft, Writing – review & editing. RN: Methodology, Writing – original draft, Writing – review & editing. S-YH: Conceptualization, Funding acquisition, Methodology, Supervision, Writing – original draft, Writing – review & editing. IAS: Conceptualization, Funding acquisition, Methodology, Writing – original draft, Writing – review & editing. W-YL: Conceptualization, Funding acquisition, Methodology, Project administration, Resources, Supervision, Visualization, Writing – original draft, Writing – review & editing.
